# Climate of the Crimea

**Published:** 1857-04

**Authors:** William R. E. Smart

**Affiliations:** late Surgeon in charge of the Naval Brigade Hospital.


### TRANSACTIONS

OF THE

### EPIDEMIOLOGICAL SOCIETY OF LONDON.

### Papers and Communications.

### A SKETCH OF THE PRINCIPAL FEATURES OF THE CLIMATE OF THE CRIMEA, AND ITS EFFECTS ON HEALTH, AS OBSERVED DURING THE FIRST YEAR OF THE OCCUPATION BY THE ALLIED FORCES.

By WILLIAM R. E. SMART, M.D., H.M.S. *Diamond;* late Surgeon in charge of the Naval Brigade Hospital.

The following sketch of the nature and peculiarities of the Crimean climate, and of its more obvious effects on health, is not offered with the same amount of confidence as belongs to remarks founded on a complete series of observations, made with accurate scientific instruments.

It professes to be simply an arrangement of casual rough notes, made in a journal, during the pressure of most important duties of a strictly professional character; and, as the writer has not met with any connected and familiar account of the phenomena of the Crimean climate, he imagines that, presented in this form, the matter may be of passing interest, if not sufficiently profound to be of scientific utility.

In the earliest passages of European history, Cimmeria, now the Crimea, was fabled to be a land involved in perpetual mists and darkness, and its inhabitants were said to be addicted to the most barbarous practices, and so inhospitable that they sacrificed all strangers to a relentless female deity.

At length some Ionian adventurers, undaunted by these fables, or by the general belief of the innavigable nature of the intervening sea, pushed boldly across, and reached a sunny shore with far receding fertile plains. Here they founded the city—Panticapæum—that exists to this day under the name of Kertch; which, owing to the excellence of its commercial position, arose from being the humble tributary of barbarous tribes, to the dignity of the seat of a kingdom; and has since been in succession a dependency of the Pontine, Roman, Byzantine, Ottoman, and Russian empires.

From the date of the Ionian visit, 650 B.C., commerce began to carry civilisation on her wings to the remoter parts of Scythia, and to return laden with the native productions of those vast plains and interminable forests.

In order to encourage intercourse, these first adventurers changed the name of the sea, from that which signified inhospitable, to Εὔξεινος, the hospitable; an idea that was again acted on with similar results, after the lapse of 2,300 years, when the "Cape of Tempests", from which the first expedition had turned back in despair, received the more propitious name of "Good Hope".

The terrors arising from ignorance being thus dispelled, the argosies of Greece sailed over the Euxine in safety; and the merchants of Athens in her brightest days, at their meetings under the porticos of the Agora, calculated on the returning season of the north-east wind, that should bring into their ports of Munychia and Phalerum the cargoes of corn that had waved on Cimmerian plains, of salted flesh of the sturgeon caught in the waters of the Tanais and the Rha, and of the skins and costly furs of wild animals, ensnared on the slopes of Hyperborean mountains, or far off in the unknown wilds of Northern Asia.

The importance of this commerce increased with the growth of the Byzantine empire; but, when the Eastern mistress of the world became the metropolis of the Ottoman empire, Cimmerian darkness again enshrouded these favoured regions, and thus the ancient fables concerning them have been almost equalled in modern days.

Before the massacre of Sinope had stained the waters of the Euxine with blood, our fleet lay in the Thracian Bosphorus, hesitating to enter a sea of which the pilotage was unknown, presuming it to be studded with undiscovered shoals, and obscured by fogs and by clouds pregnant with storms. Two years have sufficed to dispel such delusions; and Western Europe remains no longer in ignorance of these among the fairest regions of the earth, the borders of that great basin, into which the largest rivers of Europe pour their inexhaustible floods.

The labours and the triumphs of war have conduced, in this instance, to the extension of the arts of peace. Our pilots now navigate fearlessly the farthest recesses of this sea, knowing that its broad bosom is free from hidden dangers. Our merchants have become more fully acquainted with the resources of the fertile provinces extending from its shores, which, when fully developed, will render it an almost unequalled emporium for the supply of less productive regions, opening up, at the same time, a wide field for the spread of our commerce and manufactures.

The ambitious disturber has been taught how futile it is to direct her energies to the fulfilment of dreams of conquest in this direction, and has been led to change her aim to the peaceful development of the riches of her soil, and to the establishment of her great commercial interests, as the only sure mode of increasing her influence among the surrounding states.

This general good has been attained at the cost of legions, of which Britain was justly proud, whose sufferings have been wrongly attributed, in great measure, to the nature of the climate in which they were called on to undergo military labours with numbers inadequate to their task. Enfeebled by epidemic disease, they were landed in the Crimea, when winter was close at hand, to fight a skilful and unwearied enemy; and, after conquering in the field, to restrain within his fortifications this foe, possessing unlimited resources, and the advantages of winter quarters, while they themselves lay encamped in the open field, suffering the worst evils of poverty. Calamities beyond calculation naturally followed; and these may have been somewhat increased by rigors of the climate.

To assist in ascertaining how much of the disease that prevailed among our soldiers is fairly attributable to the conditions of climate in which they were placed, is the main object of these observations.

The Crimea being almost surrounded by water, and connected by means of a narrow short isthmus with a vast extent of flat country, possesses, from its conformation and contiguity, a climate that partakes of both those groups of characteristics that are contradistinguished by the terms *continental* and *insular*. These opposite characters do not, however, impress their traits continuously, so as to mark each its own seasons of the year; but, by their frequent alternations, they serve rather to stamp the climate as an irregular and inconstant one, from which it may be anticipated that the advent and course of the seasons will be found to vary much in succeeding years.

In specifying briefly the leading features of the seasons, through the first year of the occupation by the allies, and taking each season in succession, it may be said of the winter, that its mean temperature was mild, with much moisture deposited in the early part of it, to the end of December; after which there were heavy falls of snow, with low depression of the thermometer, continuous through a space of three weeks. This, which may be regarded as the climax of the winter, occurred early in January; and, in receding or in advancing from that point, there was not found any long duration of cold.

A most striking peculiarity of the winter, was the suddenness of accession of great variations of temperature, by which both animal and vegetable life were submitted with rapidity to the accelerating and reproductive influences that more properly belong to the spring, and were again acted on by the retarding influences of the depth of winter; very marked changes of this kind being completed in the course of a few hours. These were simply the interchanges of the continental and insular conditions of climate, which were felt extremely in the position occupied by the allies—the Chersonese—because the mountain barrier that shelters from the northern influences does not extend so far west.

As early as the middle of February, sometimes at noon the thermometer was observed to have risen to above 70°; the galanthus and crocus, the first offerings of a grateful soil, were seen thus early bedecking every bank; and a little later, numerous varieties of bulbous and orchidaceous plants were in flower.

The spring season, from the early part of March to the end of April, was warm; and the ground was moistened by showers that fell frequently in the day, the nights being clear, cold, and dewy. The invigorating effect of this season on the animal kingdom, was strongly exemplified in the large flights of migratory birds that tarried on these shores in their course to northern fields, in the improving condition of those domestic animals that had withstood the vicissitudes of the winter, and in the joyful spirit pervading the camp, exhibited in the early institution of athletic sports, which had been, in January, as far from the ideas of the men, as they were foreign to the circumstances of their condition.

The heat of the summer was at no time excessive, not equal to that to which our troops are accustomed when stationed at Gibraltar or Malta; but in May and June it was oppressive, from the extreme dryness of the atmosphere, with northerly winds, which absorbed every particle of moisture, and gave very few showers in return. This dry heat was much complained of, because of the great evaporation that went on from the surface of the body while it lasted, but it did not appear unhealthy; it quickened the breathing and accelerated the circulation, and was by no means prohibitive of exercise to the healthy, while it seemed not to retard the convalescence of the sick. During its continuance, at this early season, the atmosphere was clear and highly rarefied; distant objects were distinctly visible; the thermometer betrayed no unusual elevation; and the difference of the wet and dry bulbs was uniformly great.

This was the period of longest continued drought; but when the sun had reached his highest, and when, if the same conditions had increased, disease might have shewn itself among animals, and barrenness would have affected the parched soil, thunderstorms began to occur, and they were repeated at moderate intervals until the end of September. By these a more pleasant atmosphere was restored to the air, impurities were cleansed away from the surface of the ground, and the almost exhausted water sources were replenished. The summer nights were almost constantly rendered cool by moderate winds; and thus there were rarely felt that exhaustion of physical power, and that depression of animal spirits, which arise from the opposite conditions, and preclude the restorative effects of sleep.

The character of the produce of a land, and the earlier or later date of its harvests, are very general, but withal good, criteria of the average conditions of its climate. To apply this, it may be stated, that on first arriving on these shores in the middle of September 1854, it appeared significant of a moderate mean temperature of the summer, that the cereals had just then been reaped, and were but partially gathered in, while the grape was not yet ripe for the vintage; and the absence of the olive afforded evidence, that the production of oil from it does not enter into the profitable agricultural resources of a country where much of that commodity is in demand.

These facts alone would lead to one of two inferences: either that the summer of 1854 was behind the average of years, or that the climate of the south-western portion of the Crimea, in its main features and mean temperature, resembles more closely that of Belgium and the south of England, that lie five degrees north of it, on the western or oceanic confine of Europe, than the plains of Lombardy and Central France, which are situated within its own parallels.

The summer season changed imperceptibly into that of autumn; and, regarding that season as a whole, I have not known any country in which the climate is found more agreeable to the sensations. An unfailing succession of cool nights to warm clear days marked the course of the season to the end of October, when the north wind, blowing coldly at intervals, began to suggest a return to the comforts of winter garments.

The change from autumn to winter, in 1854, was as sudden as that from winter to spring; and depressed even more than the latter exhilarated, rather from continued rains, than from intense cold, attending it.

The sudden mutations of temperature to which the climate of the Crimea is liable in the winter months, were more hurtful to the health of our forces than any other of the climatic conditions. These changes of heat and cold were the sequences of the shiftings of the winds from the northerly to the southerly direction, or *vice versâ*. They were of such extreme nature as to produce a fall of 30° in the thermometer within a few hours; and even then this instrument, which is moved simply by the dynamic laws of matter, was but a feeble indicator in comparison with the sensibility of the nervous organisations of man, and of animals in general. Fortunately, the stages of very low temperature were usually of relatively short duration; but the change in the opposite direction being effected with equal rapidity, it was often found to cause injurious effects on health. The changes from high to low temperature most frequently took place in the hours of the night, while those from cold to heat very generally occurred in the day time; the former being often attended with a fall of snow, and the latter by that of rain or hail.

I have said that these great vicissitudes of temperature of the winter season, together with the frequent electric changes of the summer, are dependent on the alternations of the two principal winds, or rather on the changes of relative altitude and position of the planes of these atmospheric currents, determined by the mixed continental and insular conditions of the land; and that the configuration of its surface has a strong local influence. High mountains range along its southern coasts, and form a lofty boundary, which causes the insular conditions of climate to predominate south of its barrier over that narrow strip of coast, which resembles in its scenery, as well as in its climate, the Italian "riviera" between Nice and Genoa. To this shore the imperial family and the nobility resort, to enjoy the balmy influences of a southern clime, in villas seated amid the soft seclusion of luxuriant valleys that terminate deep ravines, or on the sunny aspects of mountain slopes that decline rapidly towards the azure depths of the sea. Northward of the mountain ridge, the whole aspect of the country and its climate, except in a few sheltered vales, is assimilated to the continental conditions of South Russia; the mountains reach the low level by graduated slopes, with but few precipices, and from their foot wide plains of a gravelly soil and clayey subsoil, productive of a scanty pasturage and of fair crops of corn, extend as far as the eye can reach. The deep river beds, with their precipitous newly cut banks, exposing loops of the bared roots of shrubs, speak plainly of the winter torrent that rushes along the channel, which, in summer, is almost dry. In summer, these treeless plains are parched by the direct rays of the sun, and a wavering glare floats over their surface; in the winter, they are sodden with the early rains, and swept across by frozen blasts from the north, and then their inhospitable surface offers none but artificial shelter to the shepherd and his flocks.

The flanks of the mountain range, of which the western was the seat of war, are obnoxious to all the inconveniences of the alternations of these opposite conditions.

I have shown, or attempted to show, in the foregoing general remarks on the seasons, in what manner the continental and insular conditions interchange with each other, to generate the remarkable instability of the climate of the Crimean Chersonese, and its obnoxiousness to sudden extreme variations in the colder half of the year. Consideration must, in the next place, be extended to the more obvious phenomena that characterise the different seasons, so as to arrive at and to explain, if possible, whatever is unusual in them.

This branch of the subject will embrace the phenomena of—

1. Atmospheric currents or winds.
2. Marine currents, or streams of sea and river water.
3. Atmospheric moisture, the varying degree of capacity for its suspension, and its forms of deposit, as influenced by the above phenomena.
4. Electric disturbance, as thunderstorms, etc.

1. *Atmospheric Currents.* As a general fact, it may be stated that the northerly current prevailed at the surface of the earth during the summer months, and that from the south through the winter; although it must be understood there were frequent interruptions in each season. By this general disposition, however, the mean temperature was greatly influenced; the north wind being in the summer season a dry warm wind in the day time, and cool and refreshing at night, but in the winter it was the cold blast; and thus it produced both extremes of temperature—the heat of summer, and the winter's cold.

The northern current, having traversed the bare plains of Southern Russia and the Crimea, varies with the season of the year, as above described: the other, coming from the warmer regions of the south, across a wide expanse of sea, is always mild and saturated with moisture; it produces an equable temperature, and the clouds it brings, being attracted by the high range of coast mountains, deposit their contents in the form of rain in summer and early winter, and of hail or snow later in that season.

The direction of the winds was the most uncertain and variable at the rainy seasons that ushered in the winter and spring. The great northerly and southerly currents were then contending, as it were, for the mastery of the approaching season, by occupation of the plane nearest the earth's surface; and it was then, especially at the commencement of winter, that violent gales of wind occurred. That of the 14th of November, 1854, followed the contour of the coast; it swept from east to west, and then from south to north, following the coast line. Its disastrous effects were first felt at the anchorage of Balaklava, then at Kamiesch, the Katscha river, Eupatoria, and Cape Tarkan, in succession, revolving from south-east to west, and then towards the north point of the compass; the wind striking in a different direction at each of the above points of its course.

In the winter months, the alteration of direction from the south to the north usually took place in the night hours, and was often attended by a fall of snow; while the alteration in the opposite direction took place in the day, and was frequently accompanied by rain or hail.

Through the summer there was a pretty regular succession of land and sea breezes on the coast; the former setting in between 10 P.M. and midnight, and lasting until after sunrise, often until 7 A.M. The distant ripple of the sea that announced the approach of the latter was usually seen, like a dark blue line on the horizon, early in the forenoon; the gentle breeze usually reached the shore before midday, to die gradually away with the setting of the sun.

These phenomena were dependent on the diurnal variations in the heat of the earth and of the sea; the former undergoing changes, while the latter preserved its uniformity.

2. *Currents of Sea and River Waters.* The Euxine must be regarded as a broad, deep, inland depression, receiving at one of its ends the streams of numerous great rivers which, flowing from the north, pour into it a volume of water that varies in quantity at different seasons, and also in temperature, in accordance with the greater or less quantity of melted ice and snow that enters into their streams. The surface of land that drains its waters into this basin, extends from the northern slopes of the Caucasus to the eastern precipices of the Swiss Alps, and from the Balkan and the Dalmatian Alps in the south, to the hills of moderate height that form the northern boundary of the great central plain of Russia. The mass of water received from this great water-shed, is more than sufficient to compensate the undoubtedly great evaporation constantly going on from the surface of the Euxine; and the surplus waters find an outlet to the southward by the Bosphorus, through which there is usually an outward stream. As the supply from the rivers is greatest in the spring and early summer, so the marine currents are strongest then.

By inspection of an ordinary chart of the surrounding provinces, it will be seen that the water flowing from the central plains of Russia through the straits of Kertch and the Dnieper-liman, is thrown to the westward by the direction of the river channels. Thus, the stream of the Don courses along the south coast of the Crimea, and strikes across to the coast of Bulgaria, south of the mouths of the Danube. The water of the Dnieper and the Boug is directed at once towards the western shore; it meets with the streams of the Dniester and Danube, and deflects them along the coast of Bulgaria, where the conjoined current is further increased by that from the Don. The entire body of water proceeding along the southern shore, a lateral portion of it escapes through the Bosphorus; while the great mass, still proceeding eastward along the coast of Anatolia, and then north-west by that of Circassia, arrives off the straits of Kertch, where it begins again to receive the fresh outpourings of the northern rivers.

It is important to observe that, in this wide circulation, the temperature of the sea must be raised by contact with the southern shores, and by the influx of the rivers from the south; and that, when this current meets with the river streams from the north, which are of lower temperature than itself, the admixture is attended with abstraction of sensible heat from the atmosphere, and the condensation of its vapour commences, which, under certain circumstances, proceeds until *sea-fogs* are formed, and hang over the surface of the mingling currents.

3. *Atmospheric Moisture.* The Crimea being of almost insular form, surrounded by waters of a rather elevated temperature, it follows that its atmosphere must contain a more than average amount of moisture, from the evaporation that is constantly going on from the sea-surface. It may be said, that the warm current of water from the south is the grand source of atmospheric moisture; and, as the capacity for vapour of the atmosphere is increased by heat, the currents of air, that accompany the marine current from the south, must have a higher capacity for it than the cooler northerly currents. This was well exemplified in the early winter—in November and December, when the relative heat of the marine current is at its highest, as there is then the smallest admixture of snow or ice water poured in by the rivers, and the south winds reach the shores, laden with moisture, to meet with the northerly currents of air that have passed over a surface of land of falling temperature; and under these circumstances there occur the only truly periodical and continued rains of the climate. Later in the winter season, when the south winds meet with the now much colder northerly currents, the precipitated moisture assumes the form of snow, small showers of which were not infrequent at night in December; but until January and February there were no falls of snow so heavy as to lie on the ground beyond a day or two.

The rains of the spring season were more temporary than those of the early winter; they rarely occupied more than a portion of the day, occurring most frequently in the forenoon; and they were seldom the product of a densely overcast sky. It was frequently noticed at this season, that local showers fell, with light southerly winds, in the hottest part of the day, while great evaporation was going on from the surface of the earth, as indicated by the wide difference of temperature of the two dry and wetted bulbs. These coincidences, at first sight somewhat paradoxical, simply showed that, the southerly current being inferior to the northerly one during the day hours, there was a decreasing capacity for vapour in its upper plane from contact with the cooler north current, simultaneously with an increasing capacity of the lower plane, caused by heat radiated from the earth's surface. From these coexisting conditions, the upper plane was condensing vapour, of which it was receiving constant additions from the ascending currents of heated air, that bore away the great evaporation from the soil, absorbed by the atmosphere nearest to its surface.

The meeting of those two main currents of air of different temperatures is certainly the prime cause of the precipitation of the atmospheric vapour in the form of rain, snow, or hail; and thus the majority of the phenomena of moisture, that occur in the colder half of the year, without any great electric disturbance, can be readily enough accounted for. But the existence of a lofty range of mountains, parallel to and not far from the southern coast, has a considerable influence on these phenomena; this influence being of greatest extent in the summer season, when the falls of rain were most frequently in thunder storms with much electric disturbance.

There are some other phenomena of moisture, such as dew and fogs, that arise from a somewhat different train of causes, and require a separate consideration. In as far as they are limited to the land, they are dependent on the diurnal variations of terrestrial heat, on the inequalities between this and that of the atmosphere, and on the clearness of the sky from clouds; while, in the formation of sea-fogs, the marine currents cooperate with those of the atmosphere, to effect condensation of vapour, by similar means of variations of temperature.

In explanation of the *dew and fogs of the land*, it must be repeated that the main supply of vapour is that brought to these shores by the south winds. Now, the capacity of the atmosphere for vapour preserves a certain uniformity as long as it is carried across the sea, of which the temperature, under ordinary circumstances, is slow to change; but, when by the onward course of the wind, it is brought into relation with the surface of the earth that undergoes great diurnal variations of heat, the capacity for vapour of the superincumbent air, and the elasticity of the vapour itself, undergo corresponding stages of increase and diminution.

From these causes it happens that, in the hot days and cool nights of spring and autumn, when south winds blow during the day and north winds during the night, as the land becomes much heated in the day, so the air becomes loaded with insensible moisture; and, as the earth's surface is cooled again at night by the passage of the northerly current, and by free radiation into the clear sky which attends it, the atmosphere then deposits its excess of vapour in the form of dew on the surface of the land, of which the temperature has been reduced below its own. In summer this process of deposit is usually restricted to the after-midnight hours, because, during the long day, the earth has absorbed much heat, to radiate which occupies a great portion of the short night; but, as the cooler season advances, it commences earlier, and thus heavy dews, almost approaching to rain in saturating effect, were observed in April and May, and again in September and October, because, from the more rapid cooling of the earth, the period of deposit then extends almost from sunset to sunrise.

As the season approximates more closely to that of greatest cold, the same conditions existing during the day, the atmospheric vapour is equally great in quantity; and, as the air becomes very cold at night, its capacity for vapour, and the elasticity of the vapour itself, are both greatly reduced; and it is then abundantly condensed into watery particles, that remain suspended in the air in the form of fog, because, the surface of the earth not having undergone an equal amount of cooling with the air, the deposit on its surface in the form of dew cannot be effected. Under these conditions, the suspended particles subside into the depressions of the earth's surface, to form fogs there, which dissolve and concentrate the emanations arising from the warmer soil. These deleterious agencies disappear under causes that restore a relatively higher temperature to the air over that of the earth; thus, by a change to a warmer wind at night, they may be deposited as dew; or they are dissipated in the morning, as fleecy clouds amid the ascending currents of air, or as a sheet rolled off to seaward, as the sun's rays acquire calefacient force. As this phenomenon depends on the soil possessing a higher relative temperature than the air, it follows that fogs are densest and of most frequent occurrence at the decline of the year, when the earth has not yet radiated all the superficial heat accumulated by absorption during the summer months, and while the atmosphere is undergoing very rapid cooling by the passage of the currents from northern latitudes.

By taking advantage of these natural phenomena, the Russians accomplished successfully the first stage of the battles of Balaklava and Inkermann, in the months of October and November. They brought their dense columns undiscovered, into close apposition with the outposts of the allies, by advancing along the deep valleys and ravines, in the conformation of the country, that were filled up with dense mists and fogs. The allied forces were not assisted by these natural phenomena in their assault on the enemy's fortifications at dawn on the 18th of June.

The season when these phenomena occur most frequently on land, differs from that when the sea fogs, that have been so much dreaded, are formed along the coasts. In the spring and early summer, these render the approach to the shores of the Crimea hazardous, and they often produce great injury when driven in over the land, by blighting the early crops. They occur only at that season, because it is only then that the rivers bring down the waters of thawed snow and ice to mix with the warm currents of the sea.

In describing the marine currents, it was shewn how the admixture took place, of a great body of warm saline water from the south, with the colder river water from the north, over the northern area of the Black Sea, commencing at the straits of Kertch and extending to the Danube, between which points lie the mouths of the rivers that drain a very wide surface of the eastern and central Europe. By this mixture of liquid masses of different temperatures, saline constituents, and densities, much of the sensible heat of surrounding media is abstracted from them, and rendered latent in the particles of the mingling currents. The atmospheric capacity for vapour is diminished so far, that precipitation in the form of watery particles is effected; and these remain suspended in the air, in consequence of the surface of the sea being warmer than the suspending medium. The condensation of these particles forms the sea-fog, which becomes the more dense in proportion as the air is less disturbed by winds.

From the frequent changes of atmospheric temperature, caused by changes of the winds, these sea-fogs rarely last more than four or five days. Their revaporisation may be effected by either of three processes:—

By increase of force of the southerly winds, through which the capacity for vapour is increased by the heat generated by motion of the atmospheric atoms; the fog being driven before it is thus partially dispelled, and the remainder, being carried in over the warmer land, is there reconverted into vapour.

By the influence of still colder currents of air from the north, that cause, by the still further abstraction of heat, the coalescence of the watery particles, which are then deposited in the form of rain.

Lastly, by the direct power of the sun's rays in penetrating the bed of condensed vapour, and rarefying it by heat, so that it is slowly lifted from the surface of the sea, and dissipated as it ascends.

4. *Electric Disturbance.* The phenomena of restoration of equilibrium were of almost nightly occurrence during the summer months, when lambent sheets of lightning were seen to play among the clouds, agitated around the Tchatir Dagh, and the ridge of the less collected coast range. These were ordinary phenomena that indicated moderate interchanges, without any high degree of electric tension, between the northerly and southerly currents of air, aided by the inductive action of the high points of land. In addition to these, there was a peculiar liability, considering the position of a country that lies about midway in the temperate zone, to storms with violent interchanges of electric fluid, in forked explosions that indicated the highest amount of electric tension: and these storms were always preceded by a few days continuance of northerly wind with a hazy cloudless sky, and a hot parching state of the atmosphere. This hot dry current being in a state of minor electric tension, meeting with the south wind, bearing clouds replete with moisture and surcharged with electricity, violent electrical action was readily elicited by friction of the opposing currents of air; and thunder storms for the restoration of the electric equilibrium, with sudden condensation of vapour in very heavy falls of rain, were produced. The most violent of these storms occurred at night, and the intervals between them rarely exceeded a fortnight. They were so local in extent, that they scarcely ever involved simultaneously the positions of the camps on the plateau, at Balaklava and at Kamiesch, that form as it were the angles of a triangle, not exceeding six miles in its longest side.

Two memorable events define, approximately, the periods within which they occurred with greatest frequency. On the 22nd of June a thunderstorm burst over the plain of Balaklava and the valley of the Tchernaia, without making itself felt in the camp on the heights of Sebastopol. Portions of the railway were broken up by it, and huts, men, and cattle were carried away by torrents that overflowed the water channels of the plain. On the 14th of September, the army landed at Old Fort, and on the night of their disembarkation their position was deluged by a heavy fall of rain; and, being without tents, the soldiers suffered very greatly, in consequence of the drenched state of their equipments, and the sodden condition of the soil.

These violent changes, although attended by more or less of local or personal injury or inconvenience, were productive of a most salutary effect, as, through their agency, the months of July, August, and September, usually the most unhealthy months in the Levant, were rendered more agreeable to the sensations than the earlier months of May and June.

About the same period as that of thunder-storms, small whirlwinds were seen to play across the low lands, raising slender columns of dust, sand, and stones, to a high elevation. They resembled, in form and appearance, the waterspouts met with at sea; and as many as four of these phenomena have been counted at one moment, progressing over the plain of Balaklava, their cloud-base dipping occasionally towards the earth, and their tube-like pendent extremity being deflected by different strata of the atmosphere, through which they travelled slowly along.

From the repeated heavy falls of rain that occurred while bombardments were actually in progress, it may be suspected that electric changes with condensation of moisture resulted, in some degree, from the ascent of heated columns of air containing gaseous particles, the products of explosions, capable of undergoing further chemical changes, and of exciting electric activity by that process.

The camp diseases of this period were fevers, of varying type, usually presenting a periodic or paroxysmal form; scorbutic cachexy, in its protean forms; cholera, diarrhœa, dysentery, and jaundice. It remains to be determined how far those diseases clearly originated in, or were aggravated by, climatic influences.

The allied forces, who landed in the Crimea in the autumn of 1854, brought with them the choleraic agencies, which had lost their epidemic intensity previously to embarkation, but had retained sufficient energy to produce many fatal cases during the voyage.

On the night of landing, the troops were exposed to a drenching rain that fell like a torrent, being one of the summer thunder-storms. During the march to Sebastopol, they lay without shelter on the ground, exposed to the night dews, so abundantly deposited at that season of the year. Of necessity they were rationed on salted meats, which excited thirst, to assuage which they found no pure water, and indeed but little of any kind; and they ate with avidity the unripe grapes that grew plentifully along their line of march. After that fatiguing morning advance, and the hard fought day of Alma, they bivouacked on the field they had won, which had been previously occupied by the encampment of the routed enemy, who, it was said, had not been free from cholera; and on the second day after the battle, the epidemic again broke out in their ranks.

In preparation for the continuation of the march on the following morning, about sixty sufferers were brought to the sea shore over night, to await removal to the transports in the offing. The renewed march, the counter march, the busy preparations for the siege, and the sustaining excitement of high hopes of a speedy favourable termination of the bombardment, served to restrain the ravages of disease, and to prevent its again assuming epidemic characters, although scattered cases were always present in the camp; and the great proportion of officers attacked at this time was very remarkable.

This incident seemed to disclose the reason of the exemption of officers from cholera in garrisons, where they use a generous diet; since, where they were undergoing the same privations as the private soldier, living on a bare diet, and subject to equal fatigue, there was to them no such immunity from epidemic disease, but rather an increase of liability to it. The observation was not confined to army officers, who had undergone marches, fought a great battle, and lain on the ground a fortnight without the shelter of tents; but it was equally exemplified among the officers of the Naval Brigade, who had come from their ships, in which cholera is not a disease of officers, uninfluenced by the above predisponents, to be subject only, in common with army officers, to unusual poverty of diet, and the use of impure water.

The causes that sustained the health of the army were greatly augmented in efficacy by the fine mild weather, that lasted up to the commencement of November. By this time, the high hopes of the result of the bombardment were destroyed, as it had produced no very evident effect on the resources or on the spirit of the enemy; and, on the other hand, the battles of Balaklava and Inkermann had made it too evident that the besiegers were themselves reduced to the defensive; and further, that a difficult position was to be maintained through a rigorous winter, which had already come on them without any preparation on their part to meet it.

Gloomy anticipations had already overspread the minds of men, when the great gale of the 14th of November diminished the resources of the army in food, clothing, and ammunition; and the sum of evils was now completed by the sudden accession of the cold and wet of winter, which destroyed the roads, and rendered the communications between the port and the camps a matter of great difficulty. Under these combined circumstances of physical deprivation and mental depression, the previously concealed effects of the long continuance of a diet deficient in many of the staminal principles of healthy food, made themselves known with redoubled energy and by sudden outbreak. In this course of events, the climate, it must be observed, played but a secondary part, as one of the developing causes, and not as the essential. From the first week of December to the first of February, the embarkation of sick at Balaklava was about one thousand a week.

The scorbutic dyscrasy had grown up, producing pains of the limbs that disabled men from active exertion, deteriorating the vital properties of the blood, and diminishing vital cohesion,—morbid changes that preceded the extravasation of blood and ulceration of the soft tissues. The constitutional powers being thus undermined, there was less resistance offered to the impulse of the common causes of disease. The diminished digestive power of the relaxed mucous membrane could no longer operate on the imperfect diet; diarrhœa of the lienteric form shewed its proofs in every direction in the camp. The defective food continuing to act as an irritant on the weakened membrane, a low form of inflammatory action, speedily running on to ulceration, attacked the intestinal surface; and, its first and principal seat being the large intestine, the disease fell under the denomination of scorbutic or camp dysentery, evidently a disease of imperfect nutrition.

The choleraic or epidemic type, with its pathognomonic collapse, became less frequent as the above form of disease advanced; but up to January there was a series of cases of its typical character. There continued throughout the season a serous diarrhœa, in which chylous globules were often seen in the evacuations. This appeared to be closely allied to cholera, leading with slower steps, but with equal certainty, to the grave, proving fatal by extensive change of structure, marked by softening of the villous coat of the small intestines.

This form of disease could scarcely be considered dependent on climatic causes. It was an epidemic that seemed not to find in the bodies of men, whose systems had undergone changes from conditions existing in the camp, that material requisite to the reception and development of its essential germs. But it was otherwise with men who arrived in the scene with unvitiated constitutional powers, as almost every new regiment that landed at Balaklava in the winter suffered from cholera with collapse very shortly after landing.

The fever cases of this period were the only kind of disease that bore the impression of causes rightly attributable to the climate or locality; although even these are capable of receiving great aggravation from the accidental causes productive of camp diseases in general. The cases were characterised by great prostration of nervous power, crusted teeth and tongue, typhomania, absence of rubeoloid or petechial eruption; and, in their course, pneumonia was a frequent complication. In all of them there were evident morning remissions of all the symptoms, with severe exacerbations as night approached. The patients were very successfully treated by regarding (as of chief importance) the periodic nature of the fever, during the nocturnal paroxysms of which the affected organ underwent an increase of its inflammatory excitement. Proceeding on these indications, quinine was administered in doses of from four to six grains, repeated every third hour from daylight to noon, as long as the accession was deferred; sometimes wine was given with the quinine; and, by this treatment, there were effected a postponement and an ultimate cessation of the periodic accessions, in which hot skin, delirium, cough, and rusty viscid sputa were the prominent symptoms that called, during the paroxysms, for the exhibition of antimonials and counterirritant applications.

In these cases of the fever of prostration with periodic exacerbation, endemic causes influenced the development of morbid phenomena—the results of poverty and want, not indigenous to soil or climate, but generated wherever like causes exist.

The months of December and January, the period of greatest moisture succeeded by that of most intense cold, saw these social causes in extreme force; and it was then, that their morbific agency was exemplified in the greatest number of cases.

In February there was a great amelioration of the weather as regards temperature, moisture, and the aspect of the sky; and simultaneously with this, there was a great amendment of the defects of diet and clothing, which effected a rapid decline of the forms of disease above noted. The adynamic type of fever disappeared, and bowel diseases decreased rapidly in frequency; and, what was a still better indication of a good sanitary condition, they were reduced to simple forms, devoid of choleraic or scorbutic features.

The absence of tubercular disease throughout this trying season is almost as significant, pathologically, as the undue frequency of other diseases; as, in a *primâ facie* view, it affords an argument in favour of the climate of the Crimea being opposed to the development of that cachexy; further experience is, however, required on this point. Some importance attaches to this, that when causes productive of general diseases, especially those of epidemic or endemic origin, or those that expend their morbific impetus on any particular set of organs, predominate, then other sets of organs possess a certain amount of immunity from diseased actions that ordinarily develope themselves in their tissues.

The improvement of general health was progressive through March; and it may be said to have reached then its highest point, as there was almost an entire freedom from the diseases that had prevailed in the winter. Towards the end of that month, and in the beginning of April, it was observed that the greatly increased solar heat during the day, co-operating with dampness of soil produced by frequent showers, brought forth a most luxuriant vegetation, and effected a very rapid decomposition of dead organic matter, so that in every part of the camp, in the cemeteries and refuse-pits, carcases that had been carelessly buried in the winter made their position known by exudations of putrid sanies that blackened the soil, and polluted the neighbouring atmosphere.

These noxious sources of unhealthy principles required several weeks for their extinction, by means of layers of charcoal, quick lime, and fresh soil; but during the period of their activity, the atmosphere being much cooled at night, mists and fogs were common phenomena; and these served to concentrate the foul miasmata, condensed together with the aqueous vapour with which they had risen from the soil.

Fevers now began to be the most frequent form of disease. Of continued type on their first invasion, they assumed sooner or later periodic types, without any marked tendency to visceral implication. There was in them a determination to the surface rather than towards internal organs; crises by sweating were of ordinary occurrence. and the treatment by tartar emetic with laudanum was found very successful in subduing the pyrexial action.

At this time there were some outbreaks of eruptive fevers —small pox and measles,—in ships of the fleet, that were beyond the range of the asthenic or miasmatic agencies operating in the camp. These latter diseases must have been brought from distant shores; they did not extend to the camp.

In the third week of April, and again in the second week of May, cholera with collapse, that had been unseen through three months, made distinct outbursts, following, on each occasion, falls of rain after continued drought, while the mean temperature was on the increase. In the camp it was most frequent, at first, among men doing the duties of the trenches; some of the earliest cases being brought out of them. Certainly there was much of refuse matter in these places, which acted as drains to the higher ground; and many corpses of the killed in the batteries had been hastily interred in the immediate vicinity. In the harbour of Balaklava it was most prevalent among the vessels lying on the town side, at the wharves that had been raised in level, through the winter, by a concrete accumulation of mud and decaying vegetable and animal matter.

It was observed again now, as in the depth of the winter, that while the disease was but little inclined to become epidemic among bodies of men that had become habituated to the specific causes, as they slowly gained force, it produced its fullest epidemic action on those brought suddenly within their influence. Our own Land Transport corps, and the army of our gallant allies, the Sardinians, suffered terribly from the disease in its very worst form. The malady, as it existed among them, was brought almost within the same category as many of the recorded outbursts among our armies in motion in India, when they have seemed to march suddenly into the very stratum or bed of localised choleraic agencies.

Although the disease appeared to have revived in the spring, under the combined action of heat and moisture, yet there was noticed to be a great and permanent amelioration immediately subsequent to the very heavy thunder-storm of the 22nd of June, from which we may date its decline. As the summer season advanced the disease abated everywhere in frequency and in severity; and with the decline of this disease, whose action is to drain the blood of its albumino-fibrine and watery particles, there was an increase of purely inflammatory affections of the bowels, mostly confined to the large intestines.

One pathological feature pervaded the autumnal diseases, viz., disordered hepatic function, marked in some cases by signs of inflammatory excitement of the organ; in some, by suppression or by reabsoption of its proper secretion, constituting jaundice; and in other cases, by the secretion of large quantities of bile of an acrid or irritating quality, that often accompanied the dysteric purging.

Thus, the diseases of the abdominal viscera were the chief organic affections of the season. The fevers of the hottest part of the summer and autumn all presented some form of derangement of the function of the liver; and, in the severest cases, they were closely assimilated to the fatal bilious-remittent fever of tropical climates.

I have thus attempted to demonstrate the most obvious peculiarities of the climate of the Crimea and to shew what were the most prevalent types of disease. It appears to me that, before we affix to these the intimate relations of cause and effect in any but the clearest instances, the full support of analogies taken from the history of similar diseases in other countries and situations, should be found to concur with our inferences, as to the climatic conditions that may have affected prejudicially the health of the allied armies; and further, that to exclude all adventitious causes of disease, which do not properly belong to the climate or locality, but which have been introduced by, or have risen among, the sufferers themselves, is especially necessary before we proceed to condemn a climate as unhealthy. To do this we require to know more of the country, and more concerning the usual sanitary conditions of the city of Sebastopol itself, previous to the siege, than we are at present acquainted with. An exact knowledge of the pathological conditions existing among the besieged would also aid very much in our estimation of the causes that gave origin and intensity to those of the besiegers.

It may be admitted that, if the expression *climatic causes of disease* is restricted to those which are exclusively atmospheric or electric, this paper will fail to bring to notice any of these agencies, as having originated the diseases of which the "Returns" are chiefly made up.

The most numerous morbid affections, according to nosological arrangement, have been, for the most part, such as are imputable to the common causes of disease, as excessive labour together with defects of shelter, diet, and clothing; or to specific essential causes, often aroused into action, and aggravated in extent and intensity by the co-operation of those common causes.

Thus the ordinary forms of *diarrhœa,* the phenomena of *disordered hepatic function,* and that simple type of *dysentery* which does not tend to ulceration of the mucous surface, are but ordinary pathological conditions, that exist under circumstances wherein the nature of the food is ill suited to the wants of the system, especially under the influence of an elevated temperature.

*Scurvy,* which gives a taint to the system that greatly aggravates all these maladies, and imparts to them worse features, arises from defective nutrition.

Climate has but a remote influence in the production and growth of any of these maladies. It may be the predisposing cause to some of them, and may rank among the "lædentia"

in all; but, of itself, it can scarcely become the exciting cause.

Again, diseases of a zymotic character, as *cholera* and *choleraic diarrhœa*, and the *low forms of fever* that exist, under fixed conditions, in all countries within certain latitudes, cannot be rightly assigned to the agencies of locality or climate as their essential cause.

*Fevers of periodic form,* remittent and intermittent, are universally admitted to be of endemic origin; and the success of medicine in their prevention and treatment is based on this principle. In the Crimea, cases of simple continued fever were rarely seen. Whatever the prominent features in the case of fever, even in those of the worst typhoid class, there was always to be observed a distinct tendency to remissions; and I cannot hesitate to accept as the cause of this, the impression of morbific agencies of local origin.

What really constitutes the specific cause which we term "malaria," is still unknown to us; and we can only regard it as one of those insensible potencies which produce constant and certain results, the mode of action of which is hidden from us.

It is not questioned that, in those districts where they are rooted evils, there are distinctly circumscribed localities in which the chances of morbific impregnation are greatly in excess of the average of the entire district; in this there is clearly observable a concentrability of the essential cause analogous to that which is found in cholera and typhus,—diseases of the zymotic class.

Again, that the intensity of *malaria,* like that of the essential causes of those other diseases, may be greatly modified by the influence of man, stands boldly forth in these facts,—that while he has it in his power, by industry, to reclaim lands which have been uninhabitable from this cause, other provinces that were once the seats of arts and sciences, abounding in all the appliances that wealth could produce, and teeming with a busy population, have, as civilization has declined, and the cultivation of the soil been neglected, become uninhabitable from the growth of this cause of insalubrity.

From this it may be inferred that malaria is not a principle inherent in the atmosphere of any locality, but is something which exists adventitiously in the soil, and is imparted by it to the atmosphere under certain conditions, and that it is capable of being increased by man's inattention to the laws of health, or of being eradicated therefrom by his labour.

To apply these remarks to the present instance, I would say that the configuration of the surface presented much of open and elevated plains; and that, for the most part, the requirements of the besiegers allowed these to be the site of the encampments; and it is admitted that, in those cases where events proved the necessity of removal of any portions of the forces from localities in which the sanitary conditions were injurious, such removal was had recourse to.

The undulations of the surface, and its gentle declivities ending in ravines, presented facilities for the shed of its waters; and although, from the shallowness of the soil, the ground, where much trodden on, in the rainy season, was quickly broken up into a state of quagmire, yet, as the soft cretaceous rock, covered only by a thin sward, absorbed much moisture, the return of a few days, or even of a few hours, served to restore to the ground a tolerable state of dryness.

There were certainly some positions that were less healthy than others; and these may be described, in general terms, as possessing a deeper soil more retentive of moisture, and seated, like avenues of amphitheatres, in little hollows or basins at the foot of hills or of rising grounds, having water sources at their lower margins.

When troops are encamped in these positions—and the necessities of an army will render this unavoidable at times—then the foci of many diseases, especially of fevers, will be found amongst them. Of this, I believe, some instances have been seen in this expedition; and, in the effort to remedy such defects, proof has been afforded that careful drainage of the soil ameliorates greatly, if it does not at once destroy, the localised causes.

Another circumstance, more strictly related to atmospheric conditions than that just alluded to, viz., the great and rapid changes of temperature so frequent in the winter, and usually attended by hygrometric changes, was a well marked cause of increased sickness.

Perhaps under ordinary aspects of life in the Crimea, when possessed of good shelter, and sufficient food and clothing, such changes of the weather would be but little felt, and not in such degree as to be productive of disease. And even as it was in this instance, with a low degree of these safeguards of health in operation, this variability of temperature and air did not appear to give rise to any new types of disease, but to augment the already existing morbific tendencies.

In conclusion, I would state the opinion entertained by me, deduced from the facts I have embodied in this paper, to be, that the climate of the Crimea ought not to be classed with those proved to be unfavourable to health; and, that whatever is shown to have acted prejudicially to health, has been of such modified influence that it would scarcely be felt under ordinary social conditions.

